# Targeting of MAPK-associated molecules identifies SON as a prime target to attenuate the proliferation and tumorigenicity of pancreatic cancer cells

**DOI:** 10.1186/1476-4598-11-88

**Published:** 2012-12-10

**Authors:** Toru Furukawa, Etsuko Tanji, Yuko Kuboki, Takashi Hatori, Masakazu Yamamoto, Kyoko Shimizu, Noriyuki Shibata, Keiko Shiratori

**Affiliations:** 1 Institute for Integrated Medical Sciences, Tokyo Women’s Medical University, 8-1 Kawada-cho, Shinjuku-ku, Tokyo, 162-8666, Japan; 2 Institute of Gastroenterology, Tokyo Women’s Medical University, Tokyo, 162-8666, Japan; 3 Department of Pathology, Tokyo Women’s Medical University, Tokyo, 162-8666, Japan

**Keywords:** SON, MAPK, RNA interference, Speckle, Cell cycle

## Abstract

**Background:**

Pancreatic cancer is characterized by constitutive activation of mitogen-activated protein kinase (MAPK). Activation of MAPK is associated with the upregulation of genes implicated in the proliferation and survival of pancreatic cancer cells. We hypothesized that knockdown of these MAPK-associated molecules could produce notable anticancer phenotypes.

**Methods:**

A RNA interference-mediated knockdown screening of 78 MAPK-associated molecules previously identified was performed to find molecules specifically associated with proliferation of pancreatic cancer cells *in vitro*. Expression of an identified molecule in pancreatic cancer tissues was examined by immunohistochemistry. *In vivo* tumorigenicity of cancer cells with stable knockdown of the molecule was assayed by using xenograft models. Flow cytometry and live cell imaging were employed to assess an association of the molecule with cell cycle.

**Results:**

The knockdown screening revealed that knockdown of *SON*, the gene encoding SON, which is a large serine/arginine-rich protein involved in RNA processing, substantially suppressed pancreatic cancer cell proliferation and survival *in vitro* and tumorigenicity *in vivo.* SON expression was higher in ductal adenocarcinomas than in cells of normal ducts and precursor lesions in pancreatic cancer tissues. Knockdown of *SON* induced G2/M arrest and apoptosis in cultured cancer cells. The suppressive effect of *SON* knockdown on proliferation was less pronounced in cultured normal duct epithelial cells. SON formed nuclear speckles in the interphase of the cell cycle and dispersed in the cytoplasm during mitosis. Live cell imaging showed that SON diffusely dispersed in the early mitotic phase, accumulated in some foci in the cytoplasm in the late mitotic phase, and gradually reassembled into speckles after mitosis.

**Conclusion:**

These results indicate that SON plays a critical role in the proliferation, survival, and tumorigenicity of pancreatic cancer cells, suggesting that SON is a novel therapeutic molecular target for pancreatic cancer.

## Background

Pancreatic cancer is a leading cause of cancer-related deaths [1,2]. Despite advancements in diagnostic and therapeutic modalities, the 5-year survival rate of patients with pancreatic cancer is less than 10% [3]. This poor prognosis elicits an urgent need for the development of effective diagnostic and therapeutic measures to improve patient survival. Molecular medicine may be able to fulfill this need, as exemplified by imatinib in the treatment of chronic myeloid leukemia [4]. Pancreatic cancer is characterized by constitutive activation of mitogen-activated protein kinase (MAPK), due to gain-of-function mutations in *KRAS* or *BRAF* and loss-of-function of dual specificity phosphatase 6 (DUSP6) [5-7]. Active MAPK translocates to the nucleus, activates transcription factors, and induces the expression of a variety of genes [8]. In a previous study, we screened the genome for downstream targets of MAPK and identified 78 molecules specifically associated with MAPK activity in pancreatic cancer cells [9]. These MAPK-associated molecules include molecules implicated in DNA replication, RNA editing, spindle formation, mitosis, signal transduction, and membrane trafficking. These biological processes play critical roles in the survival, maintenance, and proliferation of pancreatic cancer cells. We hypothesized that molecular targeting of these MAPK-associated molecules could result in notable anticancer phenotypes, as we previously observed by targeting *AURKA*[9,10]. In this study, we performed a systematic knockdown screening of MAPK-associated molecules in pancreatic cancer cells.

## Results

### Knockdown screening of MAPK-modulated genes in pancreatic cancer cells

**Figure 1:**
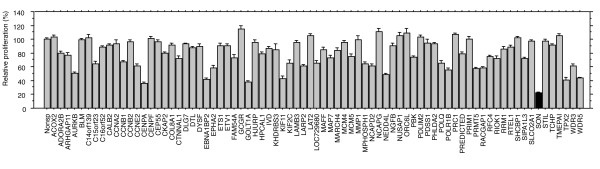
**Knockdown screening of MAPK-associated genes in pancreatic cancer cells.** Proliferation of MIA PaCa-2, a pancreatic cancer cell line, transfected with siRNA targeting various genes associated with MAPK (indicated on the horizontal axis), was determined by MTT assay on day 5 post-transfection. Plotted values are expressed relative to cells infected with control siRNA to a nonspecific sequence (Nonsp). Plots represent an average of 2 independent experiments; each experiment includes data from 8 independent transfection wells. Knockdown of *SON* (closed column) showed the most remarkable anti-proliferative phenotype.

We performed knockdown screening using a pancreatic cancer cell line, MIA PaCa-2, and custom-designed short interfering RNAs (siRNAs) targeting all the 78 MAPK-modulated genes that were previously identified and isolated in the cell line (Additional file 1: Table S1) [9]. The cells were transiently transfected with each of the 78 siRNAs, and *in vitro* proliferation was subsequently examined for 5 consecutive days. This screening showed that proliferation of cancer cells was suppressed to variable degrees depending on the individual gene targeted (Figure 1). Knockdown of *AURKB*, *CENPA, EBNA1BP2, GOLT1A, KIF11, NEDD4L, SON, TPX2*, or *WDR5* suppressed proliferation by more than 50% compared with control. Among these targets, we focused on *SON* for further study because it showed the most substantial suppressive effect. This gene encodes a nuclear speckle protein, SON, which is involved in RNA processing.

### Knockdown of *SON* attenuates proliferation in vitro, considerably in pancreatic cancer cells but less remarkably in normal phenotype cells

**Figure 2:**
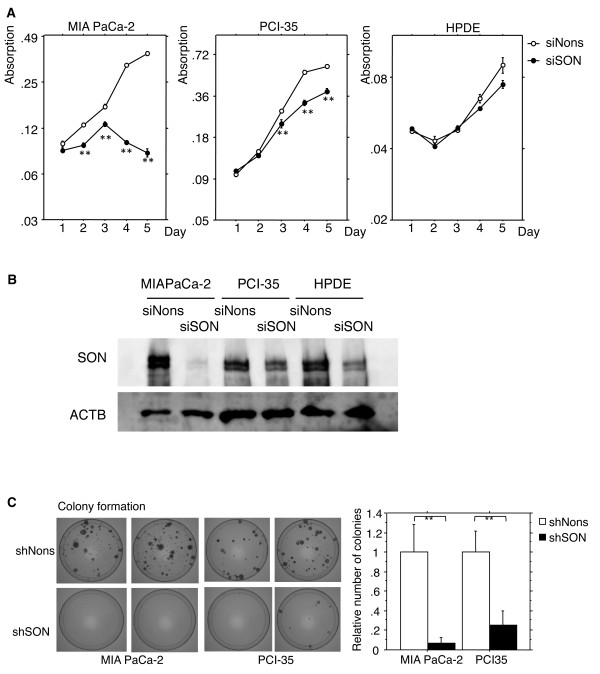
**A. Proliferation of pancreatic cancer cells (MIA PaCa-2 and PCI-35) and normally phenotypic duct epithelial cells (HPDE) transfected with siRNA against *SON *(siSON) or a nonspecific sequence (siNons) and measured by MTT assay.** The plots represent an average of 2 independent experiments; experiment includes data from 8 independent transfection wells. **B**. Expression of *SON* in cells transfected with siSON or siNons is shown in immunoblots probed with anti-SON antibody (SON) or anti-beta actin antibody (ACTB). **C**. Colony formation assay of pancreatic cancer cells transfected with vectors expressing shRNA targeting *SON* (shSON) or a non-specific sequence (shNons).

The *in vitro* suppressive effect of siRNA targeting *SON* on proliferation was reanalyzed in detail by using MIA PaCa2; PCI-35, a pancreatic cancer cell line with an aggressive phenotype; and HPDE, an immortalized normal human pancreatic duct epithelial cell line [7,11-13]. The suppressive effects of *SON* knockdown on cell proliferation appeared to be fatal in MIA PaCa-2, static in PCI-35, and insignificant in HPDE (Figure 2A). The effects of siRNA on SON expression were assayed by an immunoblotting method, which showed 77%, 10%, and 48% reduction of SON expression in MIA PaCa-2, PCI-35, and HPDE, respectively (Figure 2B). These results indicated that *SON* knockdown attenuated the *in vitro* proliferation of pancreatic cancer cells. The attenuation of proliferation depended on the efficiency of SON knockdown in pancreatic cancer cells, but was less remarkably affected in normal phenotype cells.

### Stable knockdown of *SON* reduces the survival of pancreatic cancer cells in vitro

We next constructed a vector expressing short hairpin RNA (shRNA) identical to the *SON* siRNA when processed. We examined the effect of stable knockdown of *SON* on the survival of pancreatic cancer cells *in vitro* using a colony formation assay. We found that stable knockdown of *SON* strongly attenuated the survival of cancer cells, even in PCI-35 cells, in which transient transfection of siRNA targeting *SON* modestly suppressed proliferation (Figure 2C).

### SON is overexpressed in pancreatic ductal adenocarcinomas

**Figure 3:**
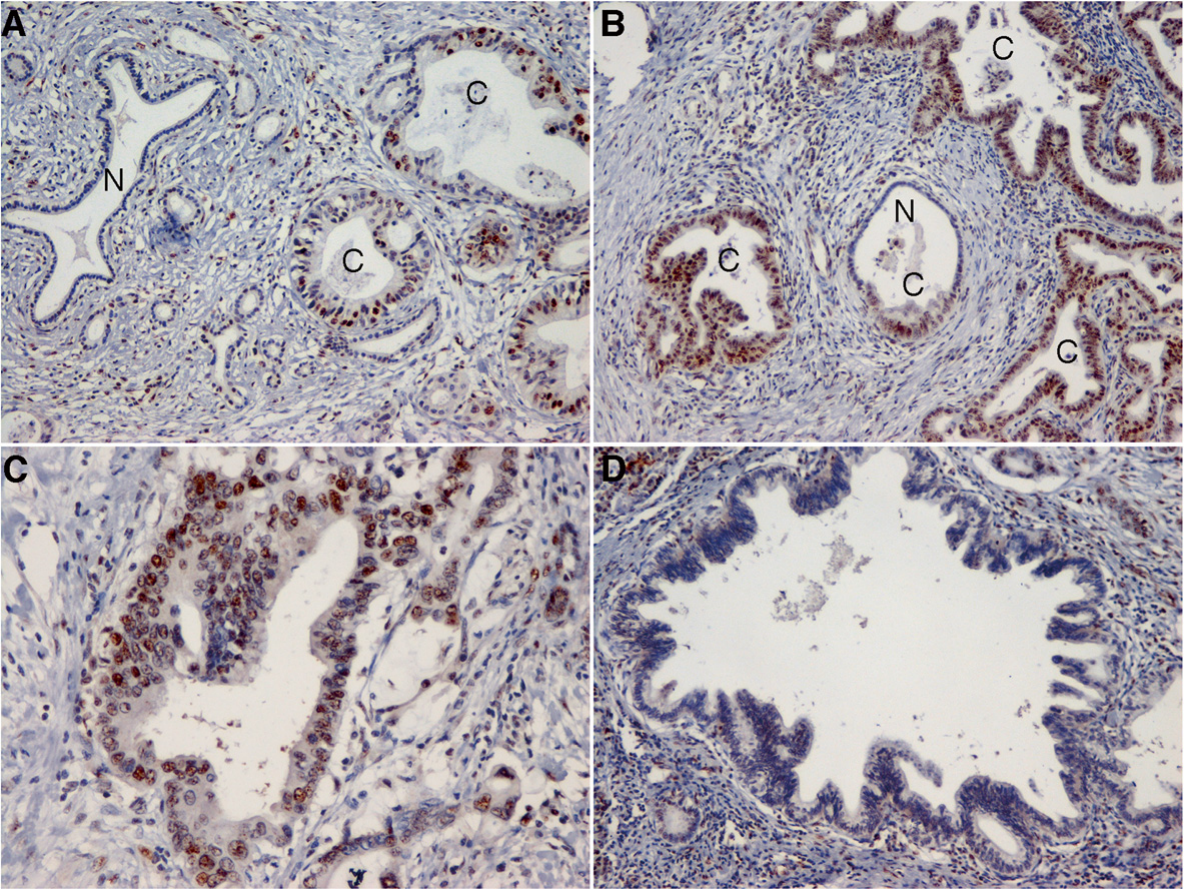
**Immunohistochemical examination of SON expression in pancreatic cancer tissues.** Diaminobenzidine and hematoxylin was used as a chromogen and a counter stain, respectively. **A** and **B**. Ductal adenocarcinomas (ducts labeled in C) strongly express SON in nuclei, more obviously than normal ductal cells (ducts labeled in N). The normal duct in panel B was partially (lower half) involved with carcinoma cells (original magnification, 100×). **C**. A high-powered view of ductal adenocarcinoma shows strong expression of SON in nuclei (original magnification, 200×). **D**. Pancreatic intraepithelial neoplasia, a precursor lesion of ductal adenocarcinoma, shows less obvious expression of SON (original magnification, 100×).

To establish the native expression of SON in pancreatic cancer, we examined 34 tissues with pancreatic ductal adenocarcinoma that were surgically resected. Immunohistochemistry showed that SON was strongly expressed in the nuclei of cancer cells in most ductal adenocarcinomas significantly more obviously than in the nuclei of non-neoplastic ducts or pancreatic intraepithelial neoplasia (PanIN), a precursor lesion of ductal adenocarcinoma (p < 0.001 by ANOVA) (Figure 3 and Table 1). This result indicates that SON is specifically overexpressed in pancreatic cancer.

**Table 1:** Expression of SON in ductal lesions evaluated by immunohistochemistry

**Ductal lesion**	**Total number of lesions**	**Intensity score**					***P*(ANOVA)**
		**1, weak**	**2, moderate**	**3, strong**			
Ductal adenocarcinoma	34	0	3	31	< 0.001
PanIN	23	15	8	0	
Normal	29	24	5	0	

### Knockdown of *SON* retards the tumorigenicity of pancreatic cancer cells in vivo

**Figure 4:**
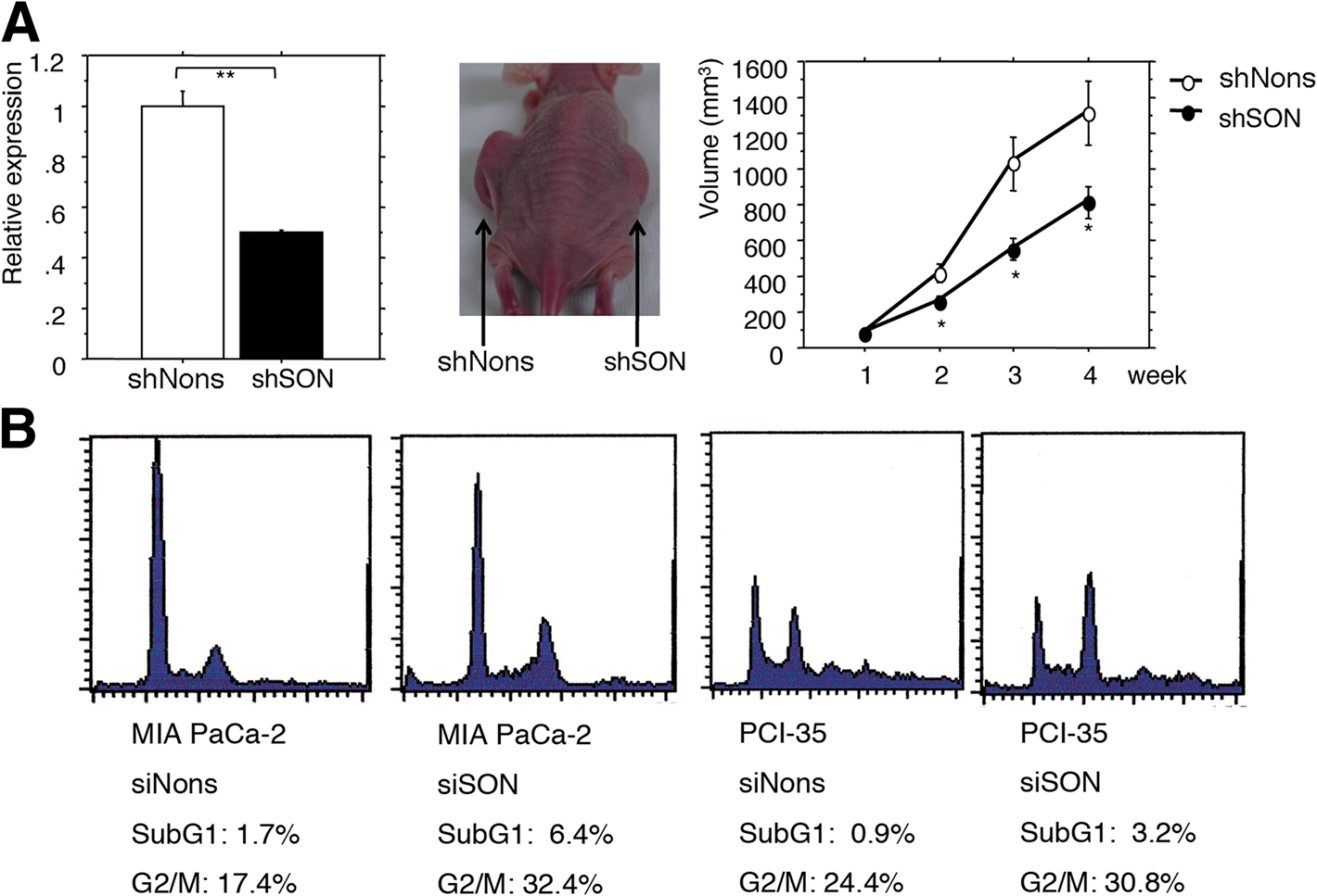
**A. Left panel: Expression of SON in cloned cells (MIA PaCa-2) stably transfected with shSON or shNons.** Middle panel: An example image of a nude mouse with a xenograft of MIA PaCa-2 clones stably transfected with shSON or shNons at 4 weeks after inoculation in the subcutis. Right panel: Average growth of tumors from xenografts of MIA PaCa-2 cells stably transfected with shSON or shNons in the subcutis of 4 nude mice. One (*) and 2 (**) asterisks indicate p < 0.05 and p < 0.01, respectively. Error bars denote 1 value of standard error. **B**. Cell cycle fractions of pancreatic cancer MIA PaCa-2 and PCI-35 cells transfected with short interfering RNA (siRNA) against *SON* (siSON) or a nonspecific sequence (siNons) as determined by flow cytometry.

We then performed a tumorigenicity assay using stably transfected pancreatic cancer cell clones carrying the shRNA vector targeting *SON*. Several stably transfected clones of MIA PaCa-2 and PCI-35 cells were obtained, and expression of *SON* was determined by real-time quantitative PCR. *SON* expression was lowest, reduced by 50%, in an MIA PaCa-2 clone (Figure 2D). We could not obtain any stably transfected PCI-35 clones in which *SON* expression was obviously reduced. This was probably because PCI-35, unlike MIA PaCa-2, could not survive modest knockdown of *SON*, which strongly suppresses the survival of cancer cells *in vitro*. The stably transfected clone of MIA PaCa-2 was inoculated into the subcutis of nude mice, and tumorigenicity was monitored. After 4 weeks, tumorigenicity was significantly retarded (Figure 4A).

### Knockdown of *SON* induces cell cycle arrest and apoptosis

To determine the mechanism by which *SON* knockdown suppresses the proliferation and survival of pancreatic cancer cells, the DNA content of siRNA-transfected MIA PaCa-2 and PCI-35 cells was measured by flow cytometry, and the cell cycle was assessed. Knockdown of *SON* increased the fraction of cells in G2/M and sub-G1, indicating that the cells were in G2/M arrest and apoptosis (Figure 4B).

### SON shuttles between the nucleus and cytoplasm depending on the cell cycle

**Figure 5:**
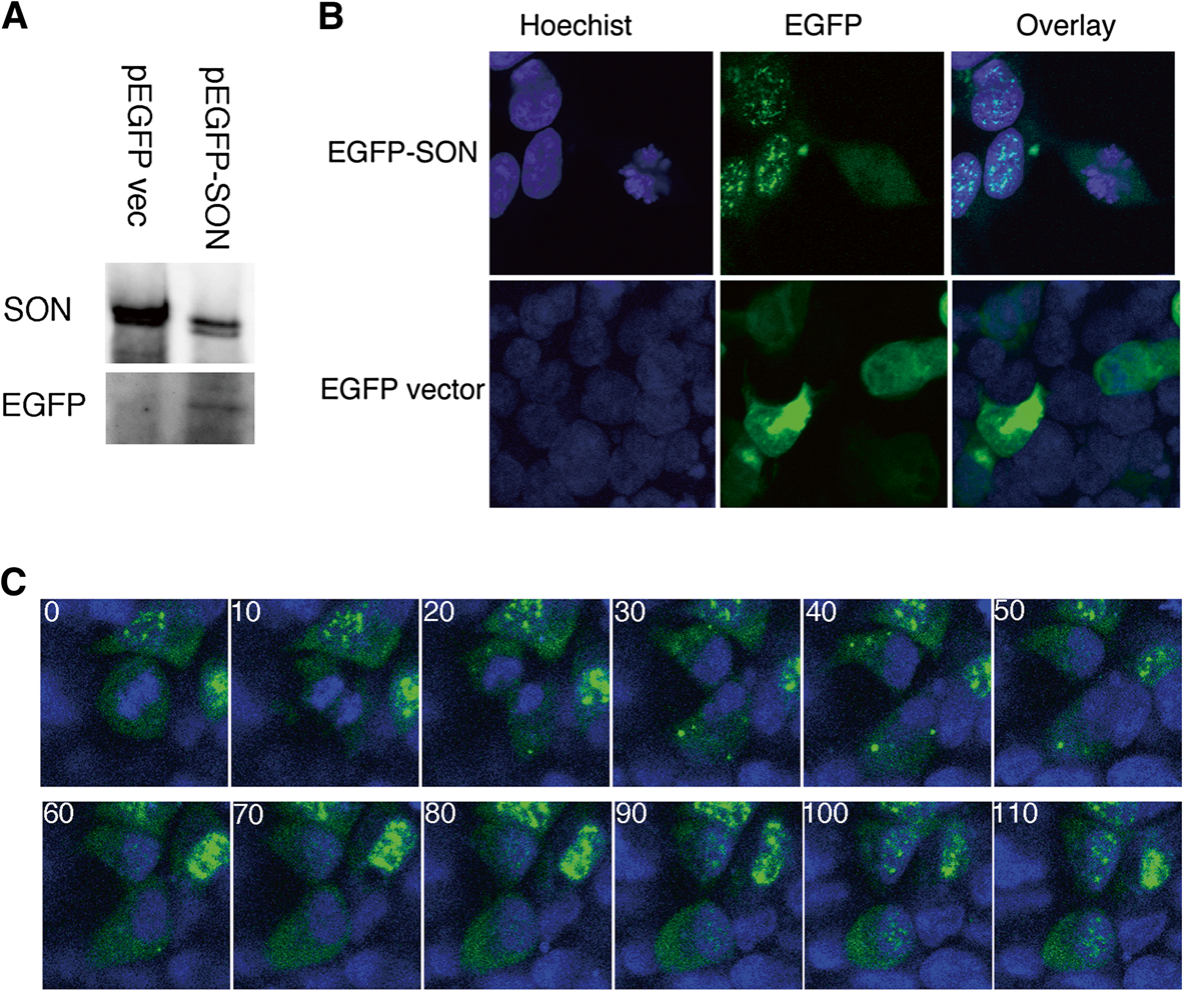
**A. Immunoblot of total cell lysate from 293 cells transfected with pEGFP vector or pEGFP-SON probed with antibodies against EGFP or SON.B**. Fluorescence images of 293 cells transfected with pEGFP-SON or pEGFP-vector. Note that EGFP-SON was speckled in the nuclei of interphase cells and diffusely dispersed in mitotic cells. Original magnification, 600×. **C**. Time-lapse images of 293 cells transfected with EGFP-SON. Sequential images taken at 10-minute intervals are shown (the number on each image indicates minutes from starting). EGFP-labeled SON dispersed in the cytoplasm during metaphase and anaphase (panels at 0 and 10 minutes), accumulated in small foci in the cytoplasm during telophase and cytokinesis (panels between 20 and 50 minutes), and then gradually reassembled in nuclear speckles (panels at 60 minutes and later). Original magnification, 400×.

To investigate the dynamics of intracellular SON expression and its role in mitosis, a vector expressing SON, tagged with enhanced green fluorescence protein (EGFP) at the amino terminus (EGFP-SON), was constructed and transfected into 293 cells. The dynamics of intracellular SON expression were then analyzed. Expression of EGFP-SON was confirmed by immunoblotting by using specific antibodies against SON or EGFP (Figure 5A). Confocal laser scanning images showed that EGFP-SON was expressed as speckles in the nuclei of cells in the interphase and was dispersed in the cytoplasm of cells in the mitotic phase (Figure 5B). Time-lapse live imaging of cells expressing EGFP-SON showed that SON dispersed diffusely in the cytoplasm in metaphase and anaphase, accumulated in some foci in the cytoplasm during telophase and cytokinesis, and gradually reassembled in nuclear speckles after cytokinesis as foci in the cytoplasm faded (Figure 5C). From metaphase, the reassembly into nuclear speckles took approximately 2 hours. These results indicate that SON shuttles between the nucleus and the cytoplasm depending on the phase of the cell cycle, transitioning from nuclear speckles and through diffuse dispersion and subsequent temporal accumulation in the cytoplasm, to slow reassembly into nuclear speckles during mitosis and the early G1 phase.

## Discussion

In this study, among many genes associated with MAPK, we found that knockdown of *SON* remarkably suppressed the proliferation, survival, and tumor formation of pancreatic cancer cells. The suppressive effect was less pronounced in normally phenotypic ductal cells. In primary pancreatic cancer tissues, *SON* was overexpressed in ductal adenocarcinomas compared with normal duct cells and PanINs. Knockdown of *SON* induced G2/M arrest and apoptosis. SON shuttled between the nucleus and cytoplasm depending on the phase of cell cycle. These results indicate that SON plays a crucial role in the proliferation, survival, and tumorigenicity of pancreatic cancer cells, thus suggesting that this molecule could be a prime therapeutic molecular target for pancreatic cancer.

Our investigation showed that knockdown of MAPK-associated molecules suppressed the proliferation of pancreatic cancer cells *in vitro* to variable degrees. We found that knockdown of *AURKB*, *CENPA, EBNA1BP2, GOLT1A, KIF11, NEDD4L, SON, TPX2*, or *WDR5* strongly suppressed the proliferation. *AURKB* encodes aurora kinase B (AURKB), which is involved in chromosome segregation and cytokinesis during mitosis [14]. *CENPA* encodes centromere protein A (CENPA), which, by functioning as a replacement for histone H3 in centromeric nucleosomes, plays an essential role in kinetochore formation and functions in cellular mitosis [15]. *EBNA1BP2* encodes a ribonucleoprotein, Epstein-Barr virus nuclear antigen 1-binding protein 2 (EBNA1BP2), which serves as a scaffold for ribosome biogenesis [16]. *GOLT1A* encodes Golgi transport 1A (GOLT1A), which functions as a transporter on the Golgi membrane [17]. *KIF11* encodes a microtubule-dependent motor protein, kinesin family member 11 (KIF11), which plays a critical role in chromosome positioning during mitosis [18]. *NEDD4L* encodes neural precursor cell expressed, developmentally down-regulated 4-like, an E3 ubiquitin protein ligase (NEDD4L) that plays a role in polyubiquitination and proteasomal destruction of SMAD2/3 [19]. *TPX2* encodes a homologue of Tpx2 of *Xenopus* (TPX2), a binding partner of aurora kinase A (AURKA) that plays a role in microtubule spindle formation [20]. *WDR5* encodes WD repeat domain 5 (WDR5), which binds methylated histone H3 lysine 4 (H3K4) and is required for recruiting H3K4 methyltransferase [21]. Among these, AURKB, CENPA, KIF11, and TPX2 are involved in functions of the microtubule spindles and kinetochores, which are considered essential for cell mitosis. Because we screened by assaying the effects of knockdown of the MAPK-associated genes on *in vitro* proliferation of pancreatic cancer cells, molecules associated with the microtubules and kinetochores might be selectively represented in our screening. Interestingly, these microtubule kinetochore-associated molecules have already been studied as molecular targets in various cancers [22-25]. Nevertheless, of these MAPK-associated molecules, we found that knockdown of *SON* most remarkably suppressed proliferation, which led us to investigate *SON* in detail as a candidate molecular target.

*SON* encodes SON, a large protein harboring a serine or arginine-rich domain. It was first cloned as a gene encoding a protein with DNA-binding activity. However, subsequently, it turned out to be a nuclear speckle protein involved in RNA processing and required for proper and efficient splicing of pre-mRNAs [26-30]. In our study, knockdown of *SON* attenuated the proliferation, survival, and tumorigenicity of pancreatic cancer cells. These suppressive effects were attributable to cell cycle arrest at the G2/M phase and apoptosis induced by depletion of SON. The association between the depletion of SON and G2/M arrest has been reported to be associated with impairment of spindle pole separation, microtubule dynamics, and genome integrity due to inadequate RNA splicing of a specific set of cell cycle-related genes with weak splice sites, i.e., splice sites without the conserved sequence [30].

Pancreatic cancer cells were more susceptible to depletion of SON than normally phenotypic cells. This may be due to rapid progression through the cell cycle in cancer cells, which results in exaggerated dependence on SON to maintain efficient RNA processing of the cell cycle-related genes. This interpretation could be endorsed by the overexpression of SON we found in most ductal adenocarcinomas, compared with normal ductal cells or precursor lesions, which suggests that adenocarcinoma cells depend on *SON* more strongly than normal ductal cells and precursor lesions to maintain their phenotypes. These results suggest that depletion of *SON* may specifically lead to an anticancer phenotype. *SON* overexpression is purportedly due to the constitutive activation of MAPK in ductal adenocarcinoma; however, other possible causes, such as gene amplification or aberrations in protein turnover, cannot be ruled out and will be a subject of further study.

The dynamics of SON distribution during the cell cycle is not well known. We performed live-cell imaging of cells expressing EGFP-SON and observed that SON dispersed in the cytoplasm during early mitotic phase formed small foci in the cytoplasm in the late mitotic phase, and gradually redistributed as speckles in the nucleus as foci in the cytoplasm faded. The cytoplasmic small foci are supposed to be mitotic interchromatin granules that correspond to accumulations of nuclear speckle proteins in the cytoplasm in the late mitotic phase [31,32]. These dynamics seem similar to the dynamics of another speckle protein, SF2, and are consistent with the idea that SON plays a role in the appropriate organization of RNA splicing factors [29,33,34].

The knockdown of SON by RNA interference showed sufficient anti-cancer phenotypes experimentally. For the RNA interference, vector-mediated stable transduction appeared to be more effective than oligonucleotide-based transient transduction as shown in Figure 2. Although the stable knockdown of *SON* by RNA interference could be an efficient molecular therapy for pancreatic cancer, the lack of a conventional method for tissue-specific, stable delivery of short, double-stranded RNA could limit the use of this approach in clinical therapeutics. Indeed, the use of RNA interference in clinical practice is generally not warranted. Recently, however, systemic delivery of siRNA combined with a special nanoparticle successfully knocked down a target gene in melanoma in a clinical trial [35]. The use of such a technique to attempt specific knockdown of *SON* in pancreatic cancer cells in a clinical model is worth trying and is an issue to be resolved in a future study. The results of this study also suggest that development of a molecule-oriented chemical substance against SON as therapy for pancreatic cancer is warranted.

## Conclusion

This study indicates that SON is overexpressed and plays a critical role in the proliferation, survival, and tumorigenicity of pancreatic cancer cells, suggesting that SON is a novel therapeutic molecular target for pancreatic cancer.

## Methods

### Cell culture

Human pancreatic cancer cell lines, MIA PaCa-2 and PCI-35, and the human embryonic kidney cell line 293 were obtained and cultured as previously described [7,9]. The immortalized human pancreatic duct-epithelial cell line, HPDE, was kindly provided by Dr. MS Tsao (Princess Margaret Hospital and Ontario Cancer Institute, Toronto, ON) and cultured as previously described [12].

### Transfection of siRNA and cell proliferation assay

siRNAs targeting each downstream MAPK-associated molecule were custom designed and manufactured (RNAi Co. Ltd., Tokyo, Japan) (Additional file 1: Table S1). Cells were seeded at 5 × 10^3^ cells/well in 96-well plates with 100 μL of appropriate culture medium and incubated at 37°C with 5% CO_2_ for 24 hours. Then, the medium was replaced with OPTI-MEM (Life Technologies, Carlsbad, CA), and the cells were transfected with siRNA at 10 nM with Oligofectamine (Life Technologies) according to the manufacturer’s recommendations. After 4 hours of incubation, the transfection reagent was replaced with the appropriate culture medium. A colorimetric cell proliferation assay—3-[4,5-dimethylthiazol-2-yl]-2,5-diphenyltetrazolium bromide (MTT) assay—was performed daily for 5 days as previously described [7].

### Colony formation assay with shRNA vectors

pSUPER vector (Oligoengine, Seattle, WA) was used for the construction of vectors expressing shRNAs by cloning the oligonucleotides 5^′^-GATCCCCGCATCTAGACGTTCTATGATTCAAGAGATCATAGAACGTCTAGATGCTTTTTA-3^′^ and 5^′^-AGCTTAAAAAGCATCTAGACGTTCTATGATCTCTTGAATCATAGAACGTCTAGATGCGGG-3^′^ to target *SON* (shRNA-SON), and 5^′^-GATCCCCGTACCGCACGTCATTCGTATTCAAGAGATACGAATGACGTGCGGTACTTTTTA-3^′^ and 5^′^-AGCTTAAAAAGTACCGCACGTCATTCGTATCTCTTGAATACGAATGACGTGCGGTACGGG-3^′^ to serve as a control harboring a nonspecific sequence against the human genome (shRNA-Nons) according to the manufacturer’s instructions. MIA PaCa-2 and PCI-35 cells were seeded at 1 × 10^5^ cells/well in 6-well plates and incubated for 24 hours at 37°C with 5% CO_2_. The shRNA-SON vector or shRNA-Nons vector were transfected into the cells with Lipofectamine^TM^ reagent (Life Technologies) according to the manufacturer’s recommendations. The cells were dissociated with trypsin 48 hours after transfection and reseeded in three 10-cm tissue-culture dishes, containing the appropriate culture medium supplemented with 10% FBS and G418 (Life Technologies) at 400 μg/mL for PCI-35 and 500 μg/mL for MIA PaCa-2. After 3 weeks, the cells were fixed with 10% formalin solution and stained with hematoxylin. The number of colonies was assessed with the COLONY program (Fujifilm Co. Ltd., Tokyo, Japan).

### Immunohistochemistry

Thirty-four formalin-fixed, paraffin-embedded tissues of pancreatic ductal adenocarcinoma that were surgically resected during 2006 and 2007 at Tokyo Women’s Medical University Hospital were studied. Indirect immunohistochemical staining was performed as previously described [36] by using a polyclonal anti-SON antibody (1:1200 dilution, Sigma, St. Louis, MO), a secondary antibody against rabbit immunoglobulin (Nichirei, Tokyo, Japan), and streptavidin solution (Nichirei). Use of the archival pathological tissues was approved by the ethics committee of Tokyo Women’s Medical University. Immunohistochemical results were evaluated among ductal lesions classified into adenocarcinoma, PanIN, or normal duct by scoring intensities of staining into 1, weak; 2, moderate; and 3, strong by comparing with normal ductal cells that showed weak staining or acinar cells that showed moderate staining. The scores were statistically analyzed by ANOVA by using PASW Statistics software (IBM Japan, Tokyo, Japan).

### Quantitative real-time polymerase chain reaction assay

The TaqMan Gene Expression Assay and a 7500 Real-time PCR system (Life Technologies) were used to analyze the transcriptional expression of *SON* by using the absolute quantitative assay according to the manufacturer’s instructions. The expression of *SON* was assessed relative to the endogenous expression of *GAPDH*.

### In vivo *tumorigenicity assay*

Pancreatic cancer cells stably transfected with shRNA vectors were isolated by cloning the surviving cells from the colony formation assay. These clones, in 50% matrigel/culture medium without FBS, were inoculated into the subcutis of BALB/c nude mice (Clea Japan Inc., Tokyo, Japan). Tumorigenicity was monitored weekly, and the tumor volume was calculated using the following formula: *V* = *D* × *d*^2^ × 0.4 (*V*, tumor volume; *D*, largest dimension; *d*, smallest dimension).

### Flow cytometry

Flow cytometric analyses for cell cycle and apoptosis were performed as previously described [7].

### Construction of the EGFP-SON vector

An expression vector containing the full coding sequence of *SON* cDNA (NM_138927) was constructed by assembling amplified products using KOD Plus DNA Polymerase and its specific buffer (TOYOBO, Osaka, Japan), appropriate paired primers, and pooled cDNA obtained from a fetal brain cDNA library (Stratagene/Agilent Technologies Inc., Santa Clara, CA) as follows. Paired primers used for amplification of cDNA fragments were C51, 5^′^-TTTAAGCTTATGGCGACCAACATCGAGCAG-3^′^ (melting temperature [Tm], 58°C) and C12, 5^′^-TAAGGGTGTTCTTGATCGCC-3^′^ (Tm, 52°C); C7, 5^′^-AGCCGCCGGAGAAGATCAAGG-3^′^ (Tm, 59°C) and C10, 5^′^-CAGGCTCTGAGGGCAAATTG-3^′^ (Tm, 53°C); and C5, 5^′^-TAAACTCAGTGAACCCAAACC-3^′^ (Tm, 50°C) and C52, 5^′^-TTTGGTACCTCAATACCTATTCAAGAAAAACATAC-3^′^ (Tm 48°C). Products amplified by PCR were sequentially cloned into the pFLAG-CMV-4 vector (Sigma, St. Louis, MO) at *Hin*dIII-*Eco*RI-*Kpn*I sites to obtain pFLAG-SON. The pEGFP-C2 vector (Clontech, Mountain View, CA) was modified by fill-in of its *Xho*I site to adjust the reading frame. The coding region of *SON* cDNA was prepared from pFLAG-SON by digestion with *Hin*dIII and *Kpn*I for the 3^′^ fragment and *Hin*dIII for the 5^′^ fragment. These fragments were sequentially cloned into the modified pEGFP-C2 vector at *Hin*dIII and *Kpn*I sites to obtain the pEGFP-SON vector. DNA sequences were confirmed by using BigDye® Terminator and a 3130x Genetic analyzer (Life Technologies).

### Immunoblot

Denatured total cell lysate was separated in a 5–15% polyacrylamide gel and blotted onto a polyvinylidene fluoride membrane by using an XV Pantera MP System (DRC Co., Ltd. Tokyo, Japan) according to the manufacturer’s recommendations. The blotted membrane was probed with anti-SON antibody (Sigma), anti-beta actin antibody (Sigma), or anti-EGFP antibody (Clontech). Horseradish peroxidase-conjugated anti-rabbit or anti-mouse immunoglobulin antibodies (GE Healthcare UK Ltd., Buckinghamshire, UK) were used for the secondary antibody reaction. Blocking conditions and concentrations of antibodies were determined according to the manufacturers’ recommendations. Signals were visualized by reaction with ECL Detection Reagent (GE Healthcare UK Ltd.) and captured digitally by using an LAS 4000 Mini (Fujifilm Co. Ltd.) or by autoradiography. Intensities of bands were measured digitally using Image Gauge software (Fujifilm Co. Ltd.).

### Laser scanning fluorescence imaging

The pEGFP-SON vector was transfected into 293 cells using Lipofectamine Plus (Life Technologies) according to the manufacturer’s recommendations. The transfected cells were incubated with Eagle’s Minimum Essential Medium (Sigma) supplemented with 10% FBS and 400 μg/mL G418. Stably transfected clones were obtained by cloning surviving cells using a cylinder cup. The isolated clones were seeded in a glass-bottom dish and incubated for 24 hours. The cells were incubated with a medium supplemented with 0.1 μg/mL Hoechst 33342 (Life Technologies) for 30 minutes. The medium was then replaced with fresh growth medium and examined under a confocal laser scanning microscope (LSM5, Carl-Zeiss Microimaging GmbH, Goettingen, Germany). Time-lapse images were obtained for 2 layers at 0- and 5-μm depth with 10-minute intervals over a total of 230 minutes.

### Statistics

Student’s *t*-test was applied to analyze statistical differences using Statview 5.0 software (SAS Institute Inc., Cary, NC, USA). P values of <0.05 were considered statistically significant.

## Abbreviations

AURKA: Aurora kinase A; AURKB: Aurora kinase B; CENPA: Centromere protein A; DUSP6: Dual specificity phosphatase 6; EBNA1BP2: Epstein-Barr virus nuclear antigen 1-binding protein 2; EGFP: Enhanced green fluorescence protein; GOLT1A: Golgi transport 1A; H3K4: Histone H3 lysine 4; KIF11: Kinesin family member 11; MAPK: Mitogen-activated protein kinase; MTT: 3-[4,5-dimethylthiazol-2-yl]-2,5-diphenyltetrazolium bromide; NEDD4L: Neural precursor cell expressed, developmentally down-regulated 4-like, an E3 ubiquitin protein ligase; Nons: Non-specific sequence; PanIN: Pancreatic intraepithelial neoplasia; shRNA: Short hairpin RNA; siRNA: Short interfering RNA; TPX2: A homologue of Tpx2 of *Xenopus*; WDR5: WD repeat domain 5.

## Competing interests

TF applied a patent on siRNAs used in this study. Other authors declare that they have no competing interests.

## Authors’ contribution

TF designed the study. TF and ET carried out *in vitro* and *in vivo* experiments and analyzed data. TF, YK, TH, MY, KShim, NS and KShir obtained, examined and analyzed surgical materials. TF wrote the manuscript. All authors had final approval of the submitted and published versions.

## Supplementary Material

Additional file 1 ** Table S1.** Short interfering RNAs used in a systematic knockdown screening of MAPK-assoicated genes in pancreatic cancer. Click here for file
