# Supplementary material for: Targeting of MAPK-associated molecules identifies SON as a prime target to attenuate the proliferation and tumorigenicity of pancreatic cancer cells
Source: Mol Cancer. 2012 Dec 10;11:88. doi: 10.1186/1476-4598-11-88 (PMC3576306; doi:10.1186/1476-4598-11-88)
Supplement: Additional file 1 — Table S1. Short interfering RNAs used in a systematic knockdown screening of MAPK-assoicated genes in pancreatic cancer. [file 1476-4598-11-88-S1.pdf]

Supplementary Table S1. Short interfering RNAs used in a systematic knockdown screening of MAPK-assoicated genes in pancreatic cancer

Accession	Gene symbol	Entretz Gene nomenclature	Sense strand	Antisense strand
NM_006109	PRMT5	protein arginine methyltransferase 5	GGAGGGACCUGAAUUGCGUCC	ACGCAAUUCAGGUCCCUCCCG
NM_001809	CENPA	centromere protein A	GGGUAUUUUUGUAGUUUCUUU	AGAAACUACAAAAAUACCCAU
NM_016343	CENPF	centromere protein F, 350/400ka (mitosin)	GGAAGCAGUAUUUGUCAGAAA	UCUGACAAAUACUGCUUCCUA
NM_006558	KHDRBS3	KH domain containing, RNA binding, signal transduction associated 3	GGUUUCCAUAGAAUUUAGUUA	ACUAAAUUCUAUGGAAACCUU
NM_006784	WDR3	WD repeat domain 3	CUUUGCACAUGAUGACAGUGU	ACUGUCAUCAUGUGCAAAGAG
NM_006824	EBNA1BP2	EBNA1 binding protein 2	CGACAGAAGCUGCAGACUAAA	UAGUCUGCAGCUUCUGUCGAA
NM_006845	KIF2C	kinesin family member 2C	CCCCGUUCCGUGAGAGCAAGC	UUGCUCUCACGGAACGGGGUG
NM_017588	WDR5	WD repeat domain 5	GAGGCCCCUUCAGUCUUGUUC	ACAAGACUGAAGGGGCCUCGC
NM_138419	FAM54A	family with sequence similarity 54, member A	GCCUACACUUGCUAUGUAAAU	UUACAUAGCAAGUGUAGGCAA
NM_000676	ADORA2B	adenosine A2b receptor	GUCACAUGCCAAUUCAGUUGU	AACUGAAUUGGCAUGUGACAG
NM_004431	EPHA2	EPH receptor A2	GCUCUGGAAUAAAGUUGCAGA	UGCAACUUUAUUCCAGAGCAG
NM_012112	TPX2	TPX2, microtubule-associated, homolog (Xenopus laevis)	GCUCAACCUGUGCCACAUUAU	AAUGUGGCACAGGUUGAGCUU
NM_014317	PDSS1	prenyl (decaprenyl) diphosphate synthase, subunit 1	GGUGCGUGGUGAAUUUCUUCA	AAGAAAUUCACCACGCACCAA
NM_014321	ORC6L	origin recognition complex, subunit 6 like	CAUGGCUUCAAAGAUACUAAA	UAGUAUCUUUGAAGCCAUGUU
NM_012323	MAFF	v-maf musculoaponeurotic fibrosarcoma oncogene homolog F	GGGAGACCGCUCUCUGCAAGG	UUGCAGAGAGCGGUCUCCCAA
NM_000160	GCGR	glucagon receptor	CCGGACACCCCCGCCAAUACC	UAUUGGCGGGGGUGUCCGGCC
NM_018204	CKAP2	cytoskeleton associated protein 2	CAAGUCCUAUUGAAAAUAUUA	AUAUUUUCAAUAGGACUUGUG
NM_013277	RACGAP1	Rac GTPase activating protein 1	CUGAUUCGAGAGAUGCUCAUG	UGAGCAUCUCUCGAAUCAGCU

NM_001033	RRM1	ribonucleotide reductase M1 polypeptide	CACCCAUUUAGCAUGCUUUUC	AAAGCAUGCUAAAUGGGUGAU
NM_002225	IVD	isovaleryl Coenzyme A dehydrogenase	GCCCUGGACGGCAUUCAGUGU	ACUGAAUGCCGUCCAGGGCUA
NM_004523	KIF11	kinesin family member 11	GAAGUGUUGUUUGUCCAAUUC	AUUGGACAAACAACACUUCGG
NM_000228	LAMB3	laminin, beta 3	CAACUUAACCUUAUGGAUUUA	AAUCCAUAAGGUUAAGUUGGA
NM_006739	MCM5	MCM5 minichromosome maintenance deficient 5, cell division cycle 46	GAACUCAAGCGGCAUUACAAC	UGUAAUGCCGCUUGAGUUCAU
NM_002421	MMP1	matrix metallopeptidase 1	GCGUGUGACAGUAAGCUAACC	UUAGCUUACUGUCACACGCUU
NM_002506	NGFB	nerve growth factor, beta polypeptide	CUGGACUAAACUUCAGCAUUC	AUGCUGAAGUUUAGUCCAGUG
NM_018454	NUSAP1	nucleolar and spindle associated protein 1	CAAUAACAAGAAUAGGGUAAG	UACCCUAUUCUUGUUAUUGUU
NM_016448	DTL	denticleless homolog (Drosophila)	CAUCCAAGUCUUUCCUGUACC	UACAGGAAAGACUUGGAUGCU
NM_032957	RTEL1	regulator of telomere elongation helicase 1	CGCUCCUGUCAUUUCUACAAC	UGUAGAAAUGACAGGAGCGAC
NM_018131	CEP55	centrosomal protein 55kDa	CCCUCUCAUUUGAUUGACAGU	UGUCAAUCAAAUGAGAGGGCU
NM_018410	HJURP	Holliday junction recognition protein	CAUAAGUUAGAUCCAAGUUGG	AACUUGGAUCUAACUUAUGGA
NM_000946	PRIM1	primase, polypeptide 1, 49kDa	CUUUCAACACCGUGAAUUUUC	AAAUUCACGGUGUUGAAAGUA
NM_018492	PBK	PDZ binding kinase	CUGUGAUGUAGGAGUCUCUCU	AGAGACUCCUACAUCACAGAU
NM_020182	TMEPAI	transmembrane, prostate androgen induced RNA	CUAUGAAUUGUACGUUUCAGA	UGAAACGUACAAUUCAUAGGG
NM_002916	RFC4	replication factor C (activator 1) 4, 37kDa	GUUCAAAAUUCCGCUUCAAGC	UUGAAGCGGAAUUUUGAACAU
NM_000057	BLM	Bloom syndrome	CGUGCGAUUUGUGAUUCAUGC	AUGAAUCACAAAUCGCACGUC
NM_022346	NCAPG	non-SMC condensin I complex, subunit G	GAGGUAUCUGAACUUAGGACU	UCCUAAGUUCAGAUACCUCAC
NM_021630	PDLIM2	PDZ and LIM domain 2 (mystique)	CAUACUAGCUCUAUAAAUAUA	UAUUUAUAGAGCUAGUAUGUC
NM_003035	STIL	SCL/TAL1 interrupting locus	CUGGAAAUUUCAUCAUAGUUC	ACUAUGAUGAAAUUUCCAGAU

NM_005630	SLCO2A1	solute carrier organic anion transporter family, member 2A1	CACAACCCCCUCUACAUGAUG	UCAUGUAGAGGGGGUUGUGGG
NM_003311	PHLDA2	pleckstrin homology-like domain, family A, member 2	GGAACCGGCUUUGAAUGAAUA	UUCAUUCAAAGCCGGUUCCCA
NM_032464	LAT2	linker for activation of T cells family, member 2	CCUAAUUGCUAUUGUUGGUUG	ACCAACAAUAGCAAUUAGGAA
NM_007088	CALB2	calbindin 2, 29kDa (calretinin)	GCUACAUCGAAGCCAAUGAGC	UCAUUGGCUUCGAUGUAGCCA
NM_024633	C14orf139	chromosome 14 open reading frame 139	CAGCUUUGGAGGAACGAUUAC	AAUCGUUCCUCCAAAGCUGGU
NM_024745	SHCBP1	SHC SH2-domain binding protein 1	GACUACCACAAUCUGUUGUCU	ACAACAGAUUGUGGUAGUCAA
NM_003494	DYSF	dysferlin, limb girdle muscular dystrophy 2B (autosomal recessive)	CUGGGGUAACGUGAAACCUGU	AGGUUUCACGUUACCCCAGGG
NM_003500	ACOX2	acyl-Coenzyme A oxidase 2, branched chain	GCAAAUGCACCCCGACAUAGA	UAUGUCGGGGUGCAUUUGCCU
NM_031480	RIOK1	RIO kinase 1 (yeast)	GUUGUACCUGCAGGUCAUUCA	AAUGACCUGCAGGUACAACUC
NM_003798	CTNNAL1	catenin (cadherin-associated protein), alpha-like 1	CUAAAGGUUGACAAGUGUAUU	UACACUUGUCAACCUUUAGAA
NM_001237	CCNA2	cyclin A2	CCCAGAAGUAGCAGAGUUUGU	AAACUCUGCUACUUCUGGGGG
NM_031966	CCNB1	cyclin B1	GUUUAUUGCAAGCAAAUAUGA	AUAUUUGCUUGCAAUAAACAU
NM_003980	MAP7	microtubule-associated protein 7	CAGGAACGAGAGAAGCAUUUC	AAUGCUUCUCUCGUUCCUGCC
NM_003981	PRC1	protein regulator of cytokinesis 1	GCUUUGAAAGAGAUGUGGUGU	ACCACAUCUCUUUCAAAGCUU
NM_004701	CCNB2	cyclin B2	GUUCCCAAAUCCGAGAAAUGG	AUUUCUCGGAUUUGGGAACUG
NM_004217	AURKB	aurora kinase B	GUAUGUAUAGGGGAAAGAAGG	UUCUUUCCCCUAUACAUACAC
NM_016195	MPHOSPH1	M-phase phosphoprotein 1	GUCUUCGAAUAAGACCAUUUA	AAUGGUCUUAUUCGAAGACAA
NM_014750	DLG7	discs, large homolog 7 (Drosophila)	GCACCUAAGGAUUUUAUGUUU	ACAUAAAAUCCUUAGGUGCGA
NM_014783	ARHGAP11A	Rho GTPase activating protein 11A	GUUUCUGAUCACAUACAGUGG	ACUGUAUGUGAUCAGAAACCU
NM_014865	NCAPD2	non-SMC condensin I complex, subunit D2	CCGGGAAGCCGUGCUUAAUGC	AUUAAGCACGGCUUCCCGGAC

NM_004702	CCNE2	cyclin E2	GUCUAUACCAUAGCAAUGUUU	ACAUUGCUAUGGUAUAGACUG
AB007899	NEDD4L	neural precursor cell expressed, developmentally down-regulated 4-like	CCUUCUUCAUUGAUCAUAACA	UUAUGAUCAAUGAAGAAGGGC
BM928826	COL8A1	collagen, type VIII, alpha 1	GGCCAAAUAUACUUUGGGUUG	ACCCAAAGUAUAUUUGGCCAC
NM_199420	POLQ	polymerase (DNA directed), theta	GUAGUAGCCAGAACUGAGAGA	UCUCAGUUCUGGCUACUACUU
AK001630	ETS1	v-ets erythroblastosis virus E26 oncogene homolog 1 (avian)	CAUCCCUUUACUUACUUGUUA	ACAAGUAAGUAAAGGGAUGUG
AB037820	MARCH4	membrane-associated ring finger (C3HC4) 4	CUGAACUAUGACAAGACAAAA	UUGUCUUGUCAUAGUUCAGCA
BU599262	GOLT1A	golgi transport 1 homolog A (S. cerevisiae)	GCUGUUCCGGAGACUUCAAGG	UUGAAGUCUCCGGAACAGCGC
AI669535	LOC729680	Hypothetical protein LOC729680	GAGUAGGAACUCUCCAACAUC	UGUUGGAGAGUUCCUACUCCG
AF070641	ETV1	ets variant gene 1	CCAUAAAAUGGGUAAUUCUCU	AGAAUUACCCAUUUUAUGGUU
AL833733	LARP2	La ribonucleoprotein domain family, member 2	CUAAAUAAACCCCAGUUCAGA	UGAACUGGGGUUUAUUUAGCU
NM_138927	SON	SON DNA binding protein	GCAUCUAGACGUUCUAUGAUG	UCAUAGAACGUCUAGAUGCUA
BC021124	C15orf23	chromosome 15 open reading frame 23	GAAGCUACUUAGGACAUCAUC	UGAUGUCCUAAGUAGCUUCCU
AK000079	LOC144703	hypothetical gene supported by AK000079	GCAUUGUUCCAUGGUUAUUAA	AAUAACCAUGGAACAAUGCCC
AK000596	HPCAL1	hippocalcin-like 1	GAACCGUGUGGGACUCACAGG	UGUGAGUCCCACACGGUUCUC
AB011117	SIPA1L3	signal-induced proliferation-associated 1 like 3	CGCAAAUCCGACGUCUUCAGA	UGAAGACGUCGGAUUUGCGGA
NM_019014	POLR1B	polymerase (RNA) I polypeptide B, 128kDa	GAUCAUACAGUGAGUGCUACC	UAGCACUCACUGUAUGAUCGG
X74794	MCM4	MCM4 minichromosome maintenance deficient 4 (S. cerevisiae)	GUUAUUCCAACUUUUGACAUG	UGUCAAAAGUUGGAAUAACUU
AF275804	LOC388279	hypothetical gene supported by AF275804	GGGUAUUCCUGUUUAUAGACU	UCUAUAAACAGGAAUACCCAA
AK021789	C16orf52	chromosome 16 open reading frame 52	GCUUUAUACCGAAAAGCAUGU	AUGCUUUUCGGUAUAAAGCAU
